# Defining the Progression of Diabetic Cardiomyopathy in a Mouse Model of Type 1 Diabetes

**DOI:** 10.3389/fphys.2020.00124

**Published:** 2020-02-20

**Authors:** Miles J. De Blasio, Nguyen Huynh, Minh Deo, Leslie E. Dubrana, Jesse Walsh, Andrew Willis, Darnel Prakoso, Helen Kiriazis, Daniel G. Donner, John C. Chatham, Rebecca H. Ritchie

**Affiliations:** ^1^Heart Failure Pharmacology, Baker Heart and Diabetes Institute, Melbourne, VIC, Australia; ^2^School of BioSciences, The University of Melbourne, Melbourne, VIC, Australia; ^3^Department of Pharmacology and Therapeutics, The University of Melbourne, Melbourne, VIC, Australia; ^4^Experimental Cardiology, Baker Heart and Diabetes Institute, Melbourne, VIC, Australia; ^5^Department of Pathology, The University of Alabama at Birmingham, Birmingham, AL, United States; ^6^Department of Medicine, Monash University, Melbourne, VIC, Australia; ^7^Department of Diabetes, Central Clinical School, Monash University, Melbourne, VIC, Australia

**Keywords:** type 1 diabetes, diabetic cardiomyopathy, cardiomyocyte hypertrophy, cardiac fibrosis, diastolic dysfunction

## Abstract

The incidence of diabetes and its association with increased cardiovascular disease risk represents a major health issue worldwide. Diabetes-induced hyperglycemia is implicated as a central driver of responses in the diabetic heart such as cardiomyocyte hypertrophy, fibrosis, and oxidative stress, termed diabetic cardiomyopathy. The onset of these responses in the setting of diabetes has not been studied to date. This study aimed to determine the time course of development of diabetic cardiomyopathy in a model of type 1 diabetes (T1D) *in vivo*. Diabetes was induced in 6-week-old male FVB/N mice via streptozotocin (55 mg/kg i.p. for 5 days; controls received citrate vehicle). At 2, 4, 8, 12, and 16 weeks of untreated diabetes, left ventricular (LV) function was assessed by echocardiography before post-mortem quantification of markers of LV cardiomyocyte hypertrophy, collagen deposition, DNA fragmentation, and changes in components of the hexosamine biosynthesis pathway (HBP) were assessed. Blood glucose and HbA1c levels were elevated by 2 weeks of diabetes. LV and muscle (gastrocnemius) weights were reduced from 8 weeks, whereas liver and kidney weights were increased from 2 and 4 weeks of diabetes, respectively. LV diastolic function declined with diabetes progression, demonstrated by a reduction in E/A ratio from 4 weeks of diabetes, and an increase in peak A-wave amplitude, deceleration time, and isovolumic relaxation time (IVRT) from 4–8 weeks of diabetes. Systemic and local inflammation (TNFα, IL-1β, CD68) were increased with diabetes. The cardiomyocyte hypertrophic marker *Nppa* was increased from 8 weeks of diabetes while β-myosin heavy chain was increased earlier, from 2 weeks of diabetes. LV fibrosis (picrosirius red; *Ctgf* and *Tgf-*β gene expression) and DNA fragmentation (a marker of cardiomyocyte apoptosis) increased with diabetes progression. LV *Nox2* and *Cd36* expression were elevated after 16 weeks of diabetes. Markers of the LV HBP (*Ogt*, *Oga*, *Gfat1/2* gene expression), and protein abundance of OGT and total O-GlcNAcylation, were increased by 16 weeks of diabetes. This is the first study to define the progression of cardiac markers contributing to the development of diabetic cardiomyopathy in a mouse model of T1D, confirming multiple pathways contribute to disease progression at various time points.

## Introduction

The incidence of diabetes mellitus is expanding as a major health issue worldwide. It is expected to exceed 640 million people by the year 2040 (Ogurtsova et al., 2017) and thus represents a major healthcare burden. Along with its increasing prevalence, diabetes was reported to be directly responsible for causing 5 million deaths in 2015 (Ogurtsova et al., 2017), thus there is an urgent need to better understand disease progression, including at the level of complications. A major consequence of diabetes is the two- to threefold heightened risk of developing cardiovascular disease (Grundy, 2004). Cardiovascular risk increases linearly with blood glucose concentrations, not surprisingly implicating hyperglycemia as a causal factor for development of cardiovascular complications (Gu et al., 2003). In particular, diabetes has been specifically linked with heart failure. Indeed, there is a significantly increased risk of cardiovascular death in people affected by diabetes between 45 and 65 years of age (Gilbert et al., 2006).

The increasing evidence linking diabetes to heart failure has led to research specifically investigating the causes of diabetes-induced cardiac dysfunction and remodeling, termed diabetic cardiomyopathy. Diabetic cardiomyopathy is defined as diabetes-associated changes in the structure and function of the heart, and specifically the left ventricle (LV), that can develop independently of hypertension and coronary artery disease (Rubler et al., 1972; Boudina and Abel, 2010; Huynh et al., 2014). Where hypertension and coronary artery disease occur, diabetic patients exhibit even greater LV dysfunction (Boudina and Abel, 2010). Diabetic cardiomyopathy presents as numerous structural and functional abnormalities, including LV diastolic (and often also systolic) dysfunction concomitant with increases in cardiac oxidative stress, inflammation, and substantial cardiac remodeling including cardiomyocyte hypertrophy, interstitial fibrosis, and apoptosis (Tate et al., 2017; Marwick et al., 2018).

There are a number of pathophysiological triggers that contribute to diabetic cardiomyopathy. Hyperglycemia has been implicated as a central driver of many of the responses seen in the diabetic heart. The progression of diabetic cardiomyopathy in rodent models of the disease has been linked to hyperglycemia-driven upregulation of NADPH oxidase and thus an increase in reactive oxygen species (ROS). This then leads to structural and functional alterations, confirming hyperglycemia as a major mediator of diabetic cardiomyopathy (Fiordaliso et al., 2001; Tate et al., 2017). These observations have been confirmed in rodent models of both type 1 diabetes (T1D) and type 2 diabetes (T2D) (Evans et al., 2003; Huynh et al., 2012, 2013; De Blasio et al., 2015; Prakoso et al., 2017; Tate et al., 2017). Diastolic dysfunction, the most common functional deficit seen in the diabetic heart, is regarded as a consequence of this increased stiffening of the heart, attributed to morphological changes such as myocardial fibrosis as well as cardiomyocyte hypertrophy (van Heerebeek et al., 2008). Other factors such as inflammation, apoptosis, and hypertrophy, as well as impairments at the level of myofilament function in the heart, may also be key drivers of development and progression of diabetic cardiomyopathy (Ren and Davidoff, 1997; Bahrami et al., 2008; Huynh et al., 2013; Tate et al., 2017).

Downstream of impaired glycemic control, another metabolic pathway linked to development of diabetic complications in the heart is the hexosamine biosynthesis pathway (HBP), and downstream of this, O-GlcNAcylation (McLarty et al., 2013; Huynh et al., 2014; Qin et al., 2017b). The HBP is an alternative fate of glucose that leads to the rapid addition [via O-GlcNAc transferase (OGT)] and removal [via β-*N*-acetylglucosaminidase (OGA)] of the sugar moiety, O-linked β-*N*-acetylglucosamine (O-GlcNAc) to many proteins (O-GlcNAcylation) resulting in a post-translational O-GlcNAc modification (Hart et al., 2007; Slawson et al., 2010; Ngoh et al., 2011; Qin et al., 2017b). Only a small percentage of glucose is thought to be shuttled through this pathway; however, this increases in the setting of diabetes and contributes to progression of diabetic cardiomyopathy by modifying the function of various proteins (Clark et al., 2003; Hu et al., 2005; Fulop et al., 2007).

Evidence suggests that the pathways and factors involved in the development and progression of diabetic cardiomyopathy are multifactorial and are still very much unknown (Marwick et al., 2018). In addition, the time course of development of the major markers that are routinely used to measure altered cardiac function and remodeling have not been determined. At present, there are currently no treatments to specifically relieve the damage and dysfunction evident in diabetic cardiomyopathy. Instead, the current treatments for diabetic patients with heart failure are the same as those without diabetes presenting with heart failure which often lack satisfactory efficacy. Therefore, there is an urgent need to better understand the contributing factors to the progression of diabetic cardiomyopathy, to enable development of more effective treatments. Thus, the present study aimed to define the progression of various contributors and/or markers leading to the development of diabetic cardiomyopathy. Having described the relative timing of each of these factors in the progression of diabetic cardiomyopathy, we are now well placed to intervene at appropriate time points to further interrogate the contributing causal mechanisms to this disorder.

## Materials and Methods

### Animals and Induction of Diabetes

All animal research was conducted in accordance with the “National Health and Medical Research Council of Australia” guidelines, and was approved by the Alfred Research Alliance (ARA) Animal Ethics committee (AEC Ethics approval#: E/1535/2015/B). All mice were bred and housed in the ARA Animal Centre and maintained under a 12-h light/dark cycle with up to four littermates per cage. A flowchart for the reporting of animal use, number per group, and analysis in preclinical studies based on the CONsolidated Standards of Animal Experiment ReporTing (CONSAERT) template proposed by Drucker (2016) has been included (Supplementary Figure S1).

Mice were randomly assigned to either the non-diabetic or diabetic experimental groups and further randomized into endpoint age groups prior to any experimental procedures. The number of animals in each group at endpoint can be found in Table 1 and in Supplementary Figure S1. Diabetes was induced in 6-week-old male FVB/N mice via streptozotocin (55 mg/kg in citrate vehicle i.p. for 5 days; controls received citrate vehicle) for separate groups of mice which were followed for either 2, 4, 8, 12, or 16 weeks of diabetes). For each group of mice (2, 4, 8, 12, or 16 weeks of untreated diabetes), endpoint LV function via M-mode and Doppler echocardiography was determined prior to measurement of markers of diabetic cardiomyopathy.

**TABLE 1:** Systemic characteristics of diabetic and non-diabetic mice at endpoint.

**Systemic characteristics**	**Non-diabetic**					**Diabetic**				
**Time after diabetes induction (weeks)**	**2**	**4**	**8**	**12**	**16**	**2**	**4**	**8**	**12**	**16**
*n*	*8*	*10*	*9*	*9*	*11*	*16*	*9*	*10*	*11*	*10*
Body weight (g)	27.30.8	27.10.6	26.30.6	31.21.1	30.91.0	26.30.7	26.30.4	25.60.5	29.11.0	29.20.6
Heart (mg)	123.92.5	121.11.7	120.72.1	138.83.2	137.12.8	116.13.0	117.12.2	114.12.6	133.14.2	134.63.1
Left ventricle (mg)	87.91.9	84.01.3	84.31.3	91.92.6	98.22.8	83.22.8	82.31.6	78.02.0	88.02.9	92.62.2
Right ventricle (mg)	20.81.3	21.50.4	22.31.0	29.71.1	21.30.7	17.50.6*	19.30.6	20.71.1	25.91.7*	20.50.8
Atria (mg)	9.60.8	8.90.7	9.70.5	10.30.5	12.30.8	9.60.3	8.70.4	10.30.8	11.80.9	14.31.0*
Liver (mg)	145955	139630	137528	158175	150261	169255*	173755*	169854*	1789105*	195566*
Kidney (mg)	187.26.3	166.13.6	183.27.8	205.78.1	191.95.4	202.56.1	217.99.6*	232.98.2*	230.010.0*	262.38.8*
Spleen (mg)	104.42.0	95.62.6	91.01.8	102.52.8	98.32.5	119.93.8*	110.86.0*	104.05.5*	109.86.8	108.63.3
Muscle (gastrocnemius) (mg)	146.73.1	150.13.7	152.12.1	166.35.6	165.44.6	138.74.7	140.74.4	135.46.2*	153.75.9	153.16.1
Tibia length (mm)	16.60.2	16.80.1	16.80.1	17.30.2	17.60.1	16.60.1	16.90.1	17.00.1	17.60.2	17.60.1
*n*	*8*	*10*	*9*	*9*	*11*	*10*	*8*	*8*	*9*	*7*
Heart rate (bpm)	37210	33415	3319	3298	38313	37615	36917	36517	34515	37419
*n*	*0*	*10*	*9*	*9*	*6*	*0*	*9*	*10*	*9*	*5*
Water consumption (ml)	−	3.40.7	3.50.7	3.10.7	2.10.7	−	14.22.4*	16.63.1*	12.03.9*	14.45.9*
Food consumption (g)	−	3.60.2	3.90.3	3.20.3	3.10.7	−	5.30.2*	5.70.4*	4.30.5*	5.60.6*
Urine output (ml)	−	0.60.1	0.30.1	0.80.3	0.60.1	−	13.32.3*	13.62.9*	9.13.3*	12.35.3*

Morphological measurements of tissues, heart rate from endpoint echocardiograph, and metabolic cage data (2 week group was not measured) from diabetic and non-diabetic mice. Data are presented as mean ± SEM and analyzed using a two-way ANOVA with Benjamini and Hochberg *post hoc* test. *P < 0.05 vs age-matched non-diabetic mice. Italicized values are number of animals per group.

### Systemic Measurements

Fortnightly and endpoint collection of blood from diabetic mice was used for evaluation of hyperglycemia (≥26 mM), and hence onset of T1D. Saphenous vein bleeds were conducted fortnightly following STZ or citrate vehicle administration and glucose was measured using a glucometer (Accu-check Advantage; Roche, Switzerland). At endpoint, whole blood collected via cardiac puncture was assessed for glycated hemoglobin (HbA1c) using a Cobas b101 POC system (Roche, Basel, Switzerland) and differential blood cell count using a HEMAVET^®^ Hematology Analyzer 950 (Drew Scientific, Miami Lakes, FL, United States). Plasma TNFα and IL-1β were measured using mouse-specific colorimetric assay kits^1^ (Melbourne, Australia) in a subset of animals from each group. Water and food consumption and urine output were measured using metabolic cages over a 24 h period (this was not measured for mice in the 2 week group and only a subset in the 16 week group).

### LV Function (M-Mode and Doppler Echocardiography)

M-mode and Doppler echocardiography were undertaken at baseline (data not shown) and at endpoint within each group. All M-mode and Doppler echocardiography measurements were scrutinized by a previously validated internal quality control process for echocardiography in mice (Donner et al., 2018). Mice were anesthetized (cocktail of i.p. ketamine, xylazine, and atropine, KXA: dosage of 80:8:0.96 mg/kg, respectively). Echocardiography was performed using a Philips iE33 ultrasound system with 15 MHz linear (M-mode) and 12 MHz sector (Doppler) transducers. Parameters measured from M-mode echocardiography included LV anterior and posterior wall thickness and LV end-diastolic and end-systolic dimension which were used to calculate fractional shortening (FS), a measure of LV systolic function. Diastolic transmitral LV inflow images were obtained from apical four-chamber views using color flow mapping-guided pulsed-wave Doppler and were used to measure early (E) and late (atrial, A) peak filling blood flow velocities (and calculate E/A ratio), isovolumic relaxation time (IVRT), and deceleration time (all common markers of LV diastolic function). All echocardiographic images were analyzed blinded using RadiAnt DICOM Viewer (v4.6.9) software and then checked blinded for independent validation by the Preclinical Cardiology Microsurgery and Imaging Platform at the Baker Heart and Diabetes Institute.

### Tissue Collection

At endpoint, blood was collected via cardiac puncture from anesthetized mice (KXA: dosage of 100:10:1.2 mg/kg) and the heart was removed and weighed. The top third portion of the LV was fixed in 10% neutral-buffered formalin (Australian Biostain, Melbourne, Australia), followed by paraffin-embedding. The middle portion of the LV was fresh-frozen in Tissue-Tek^®^ optimal cutting temperature (OCT) compound (Tissue-Tek, Torrance, CA, United States) for immunofluorescent detection of macrophages. The remainder was snap frozen for determination of gene expression and protein content, as described previously (Qin et al., 2017a). A hind leg was removed for determination of tibia bone length to normalize heart and other organ weights (Huynh et al., 2012).

### Histology

Fixed LV sections were paraffin-embedded and sections cut (4 μm) using a microtome (Leica Microsystems, Germany). Slides were stained with hematoxylin and eosin (H&E) and cardiomyocyte width was determined by measuring across the shortest cross-sectional axis, while cardiomyocyte area was measured around the perimeter, of individual cardiomyocytes (400X magnification, five to six images, and minimum 100 individual myocytes per heart). Cardiomyocyte width and area were analyzed blinded using ImageJ (Version 1.51w, National Institute of Health, United States) (Huynh et al., 2010; Ritchie et al., 2012). Picrosirius red staining was used to measure LV collagen deposition. Slides were dewaxed and stained with 0.1% picrosirius red solution (Picric acid, Fluka, Buchs, Switzerland; pH 2.0). Collagen stained an intense red color and was analyzed blinded using ImageJ as a ratio of collagen to total image area (20X magnification, 10 fields per image), as described previously (Huynh et al., 2010; Ritchie et al., 2012).

### Macrophage Staining by Immunohistochemistry

OCT-embedded LV tissues were sectioned at 6 μm for measurement of CD68+ cells by immunofluorescence analysis. LV sections were incubated with a 1:200 dilution of CD68+ primary monoclonal antibody (AbD Serotec, Raleigh, NC, United States) and then incubated with a 1:200 dilution of Alexa Fluor 546 secondary antibody (Invitrogen, Carlsbad, CA, United States) to assess macrophage infiltration and with 0.001% Hoechst 33342 (Invitrogen, Melbourne, VIC, Australia) to stain for nuclei. Images were captured with a fluorescence microscope (Zeiss Axio Observer Z1, Germany) under 200X magnification. Using Zeiss software, macrophages were counted manually as the overlay of CD68+ and DAPI fluorescence. CD68+ positive cells in 10 images per LV were averaged and calculated per square millimeter of the section (Qin et al., 2017a).

### Apoptosis Using CardioTACS (DNA Fragmentation)

Apoptosis was characterized in de-waxed paraffin-embedded LV sections using a CardioTACS *in situ* Apoptosis Detection Kit (Trevigen, Gaithersburg, MD, United States). Positively stained apoptotic cells were stained blue while negatively stained cells were counterstained with Nuclear Fast Red. Apoptotic cells were quantified as a percentage of non-apoptotic cells and expressed as fold change from age-matched citrate control mice (20X magnification, 10 fields per image) (Huynh et al., 2012; Prakoso et al., 2017).

### Analysis of Gene Expression

RNA was extracted from frozen LV and reverse transcribed as previously described (Huynh et al., 2010). Cardiac gene expression of pro-hypertrophic markers β-myosin heavy chain (β*-mhc*), *Nppa* (atrial natriuretic peptide), the pro-fibrotic markers *Ctgf* (connective tissue growth factor), periostin (*Postn*) and *Tgf-*β, *Nox2*, *Cd36*, the macrophage marker *Cd68*, the rate-limiting enzymes for hexosamine biosynthesis, glutamine:fructose-6-phosphate amidotransferase (*Gfat*) 1 and 2, and O-GlcNAc-transferase (*Ogt*) and O-GlcNAc-ase (*Oga*), and the housekeeper 18S, were determined via real-time PCR using SYBR^®^ Green (Applied Biosystems, Scoresby, VIC, Australia) using primers generated from murine sequences. Quantitative real-time analysis was performed as previously described using the ΔΔC_t_ method to detect relative fold differences of diabetic compared with non-diabetic mice (De Blasio et al., 2015, 2017; Prakoso et al., 2017).

### Analysis of Protein Expression

In a subset of samples from each time point, western blots were performed for analysis of OGT, OGA, and O-GlcNAc protein abundance. To allow for measurement of protein levels in the HBP, frozen samples of heart tissue (∼30 mg) were homogenized in lysis buffer (10% w/v) containing T-PER (Pierce Rockford, ThermoFisher Scientific), protease inhibitor (PICx, 5%), sodium orthovanadate (1 mM), sodium fluoride (20 mM), and PUGNAc (40 μm, Toronto Research Chemicals, Canada), followed by 60 min of lyzing on ice. Samples were then centrifuged at 14,600 *g* for 15 min at 4^o^C and the supernatant was collected. Protein concentration was measured as previously described (De Blasio et al., 2015). Samples were frozen at −80^o^C for later analysis. Diluted protein lysates (60 μg) were separated on 7.5% gels followed by transfer onto PVDF membranes. Membranes were probed with O-GlcNAc (CTD110.6) mouse antibody (#9875, Cell Signaling Technology, United States; 1:1000 dilution), which specifically recognizes endogenous levels of O-GlcNAc on proteins in β-O-glycosidic linkage to both serine and threonines. The secondary antibody used was a goat anti-mouse (H + L) IgG HRP conjugate (#170-6516, Bio-Rad Laboratories, United States; 1:2000 dilution). The membrane was stripped and probed for OGT (anti-OGT, #O6264, Sigma–Aldrich) antibody with a band detected at 110 kDa. The membrane was stripped again and probed for OGA (anti-OGA (NCOAT), #sc-376429, Santa Cruz) antibody with a band detected at 130 kDa. Total O-GlcNAc (measured using total lane volume) and OGT and OGA protein expression were corrected for β-actin (8H10D10, #3700, Cell Signaling Technology, United States; 1:1,000, 45 kDa). Results of each western blot were analyzed using Image Lab (version 5.2.1 build 11, Bio-Rad Laboratories).

### Statistical Analysis

Results are presented as mean ± SEM. Data were graphed and analyzed using GraphPad Prism 8.01 software. Two-way ANOVA followed by Benjamini and Hochberg *post hoc* test was used to identify differences between groups. Statistical significance was assumed at *P* < 0.05.

## Results

### Absolute Measures of Systemic Characteristics at Endpoint

**FIGURE 1:**
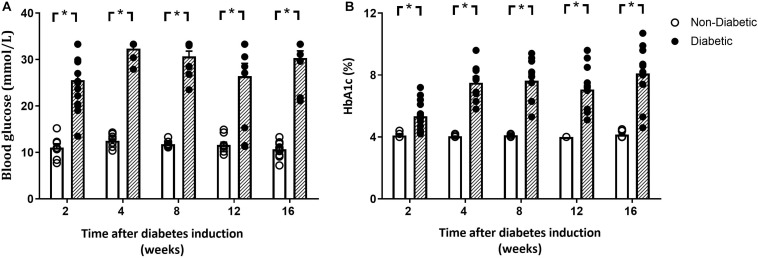
Blood glucose and glycated hemoglobin. Both blood glucose **(A)** and glycated hemoglobin **(B)** levels were elevated at each time point. Data are presented as mean ± SEM and analyzed using a two-way ANOVA with Benjamini and Hochberg *post hoc* test. *P < 0.05 vs age-matched non-diabetic mice. *n* = 8–16/group (refer to Supplementary Figure S1).

Blood glucose and glycated hemoglobin were significantly increased with diabetes at every endpoint time over the study (Figures 1A,B). Final body weight was lower in mice with diabetes overall (*P* = 0.017) and tended to be reduced after 12 weeks of diabetes on *post hoc* analysis (*P* = 0.06) (Table 1). Absolute heart weight was reduced overall by diabetes (*P* = 0.009) and tended to be reduced after 2 weeks of diabetes (*P* = 0.06, Table 1). LV weight was reduced overall by diabetes (*P* = 0.005) and tended to be reduced after 8 weeks of diabetes on *post hoc* analysis (*P* = 0.09). The right ventricle weight was reduced overall by diabetes (*P* = 0.002) and was significantly reduced after 2 and 12 weeks of diabetes, with a tendency to be reduced after 4 weeks of diabetes (*P* = 0.09, Table 1). Atrial weight was significantly elevated after 16 weeks of diabetes (Table 1). Liver weight was increased overall by diabetes (*P* < 0.001) and was significantly increased at every endpoint (Table 1). Kidney weight was increased overall by diabetes (*P* < 0.001) and was significantly increased from 4 weeks of diabetes (*P* < 0.05 for all, Table 1). Spleen weight was increased overall by diabetes (*P* < 0.001) and was increased at 2, 4, and 8 weeks of diabetes on *post hoc* analysis (*P* < 0.05 for all), with a similar tendency after 16 weeks of diabetes (*P* = 0.08, Table 1). Gastrocnemius muscle weight was reduced overall by diabetes (*P* = 0.006) and was reduced after 8 weeks (*P* < 0.05), again with a similar tendency after 12 weeks of diabetes (*P* = 0.09, Table 1). Water and food consumption and urine output, measured with 24 h metabolic cages, were all increased overall by diabetes (*P* < 0.001) and individually from 4 weeks of diabetes (Table 1). The organ weights relative to body weight are shown in Supplementary Figure S2. Kidney, liver, and spleen weights relative to body weight were increased with diabetes at each time point, with atrial weight increasing from 12 weeks of diabetes (Supplementary Figure S2).

### Measures of Systemic Characteristics at Endpoint Relative to Tibia Length

**FIGURE 2:**
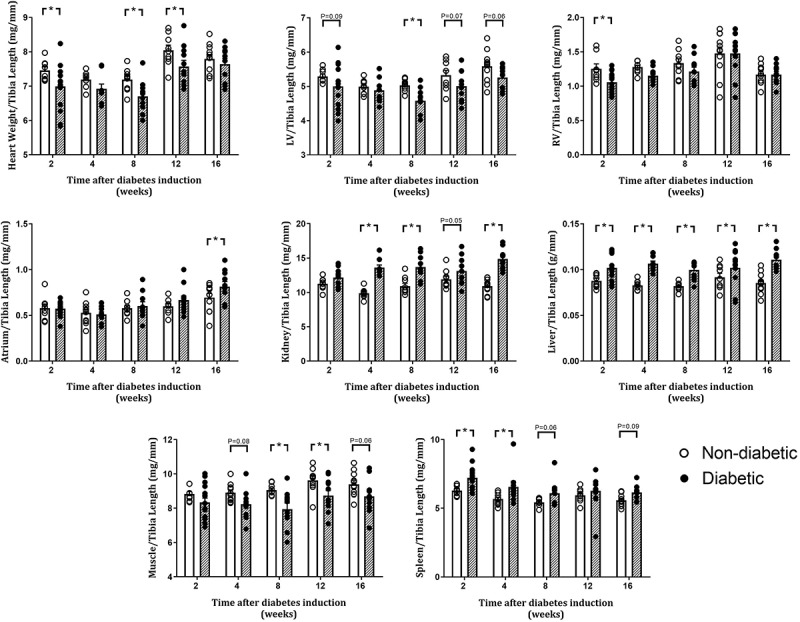
Organ weights relative to tibia length at endpoint. Data are presented as mean ± SEM. *P < 0.05 vs age-matched non-diabetic mice (two-way ANOVA with Benjamini and Hochberg *post hoc* test). *n* = 8–15/group (refer to Supplementary Figure S1).

Tibia length was unaltered by diabetes in each group at endpoint (Table 1). Heart weight relative to tibia length was reduced overall by diabetes (*P* = 0.002) and was significantly reduced at 2, 8, and 12 weeks of diabetes on *post hoc* analysis (Figure 2). Relative LV weight was reduced overall by diabetes (*P* = 0.002), was significantly reduced after 8 weeks on *post hoc* analysis, and tended to be reduced after 2, 12, and 16 weeks of diabetes (*P* = 0.09, 0.07, and 0.06, respectively, Figure 2). Relative right ventricle weight was reduced overall by diabetes (*P* = 0.02) and was reduced after 2 weeks of diabetes (Figure 2). Relative atrial weight was unaltered overall by diabetes (*P* = 0.12) but was significantly increased after 16 weeks of diabetes on *post hoc* analysis (Figure 2). Relative kidney weight was increased overall by diabetes (*P* < 0.001) and was increased after 4, 8, and 16 weeks of diabetes on *post hoc* analysis, while liver weight was increased overall by diabetes (*P* < 0.001) and was increased from 2 weeks of diabetes (*P* < 0.05, Figure 2). Relative gastrocnemius muscle weight was reduced overall by diabetes (*P* < 0.001) and at 8 and 12 weeks of diabetes (*P* < 0.05), while spleen weight was increased overall by diabetes (*P* < 0.001) and after 2, 4, and 8 weeks of diabetes (Figure 2).

### Diastolic and Systolic Function

**FIGURE 3:**
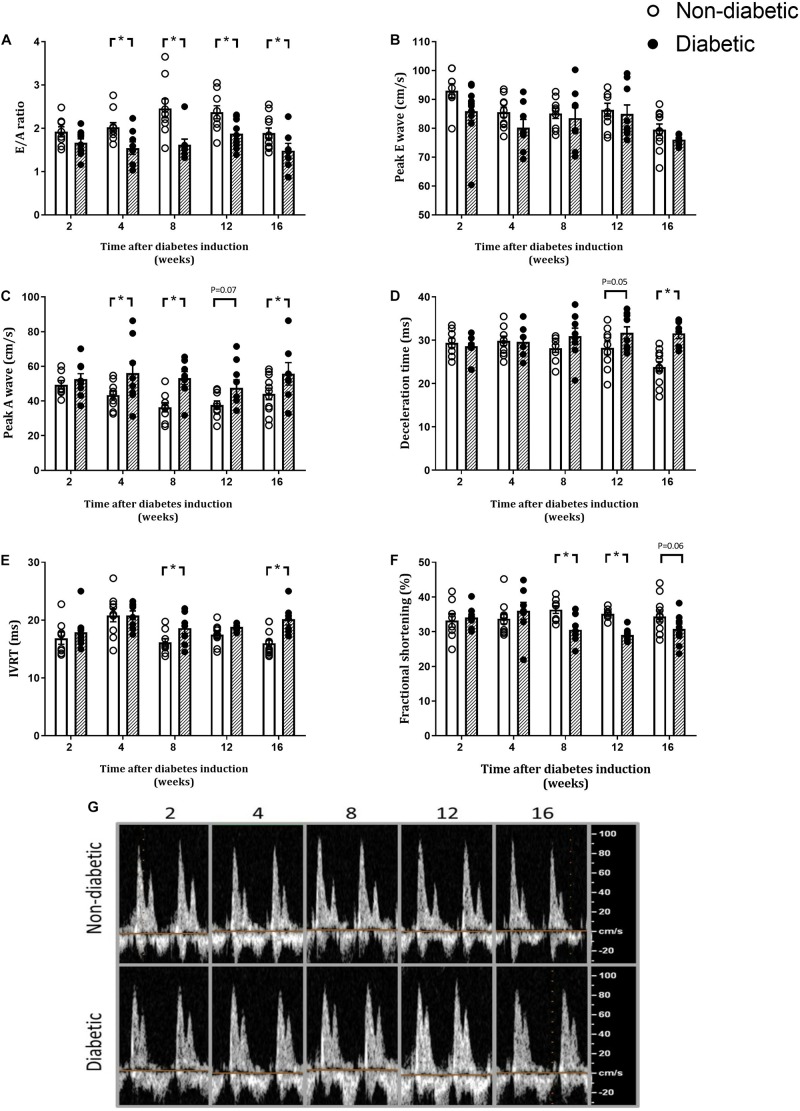
Diabetes progressively worsens markers of diastolic and systolic function. E/A ratio **(A)**, peak E wave **(B)**, peak A wave **(C)**, Deceleration time **(D)**, IVRT **(E)**, fractional shortening **(F)**, and representative doppler images **(G)** are shown. Data are presented as mean ± SEM. *P < 0.05 vs age-matched non-diabetic mice (two-way ANOVA with Benjamini and Hochberg *post hoc* test). Representative images for each endpoint Doppler echocardiograph are shown. *n* = 7–11/group (refer to Supplementary Figure S1).

Heart rate during echocardiography was unaltered with diabetes overall and at each time point (Table 1). The E/A ratio, a marker of LV diastolic function, was reduced overall by diabetes (*P* < 0.001). In addition, the E/A ratio was significantly reduced in the diabetic group at each time point from 4 weeks of diabetes on *post hoc* analysis (*P* < 0.05) (Figure 3A). Peak E wave velocity was reduced overall by diabetes but not at any individual time point (Figure 3B). Peak A wave velocity was increased overall by diabetes (*P* < 0.001) and also increased from 4 weeks of diabetes on *post hoc* analysis (*P* < 0.05) (Figure 3C). Deceleration time was increased overall by diabetes (*P* = 0.001) and was increased from 12 weeks of diabetes on *post hoc* analysis (*P* < 0.05) (Figure 3D). IVRT was increased overall by diabetes (*P* = 0.001), and on *post hoc* analysis at 8 and 16 weeks of diabetes (Figure 3E). FS was reduced overall by diabetes (*P* = 0.007) and on *post hoc* analysis was reduced from 8 weeks of diabetes (*P* < 0.05) (Figure 3F). Echo data for the above measures are also presented normalized to each age-matched non-diabetic control group (Supplementary Figure S3).

### Circulating Inflammatory Markers and Macrophage Infiltration

**FIGURE 4:**
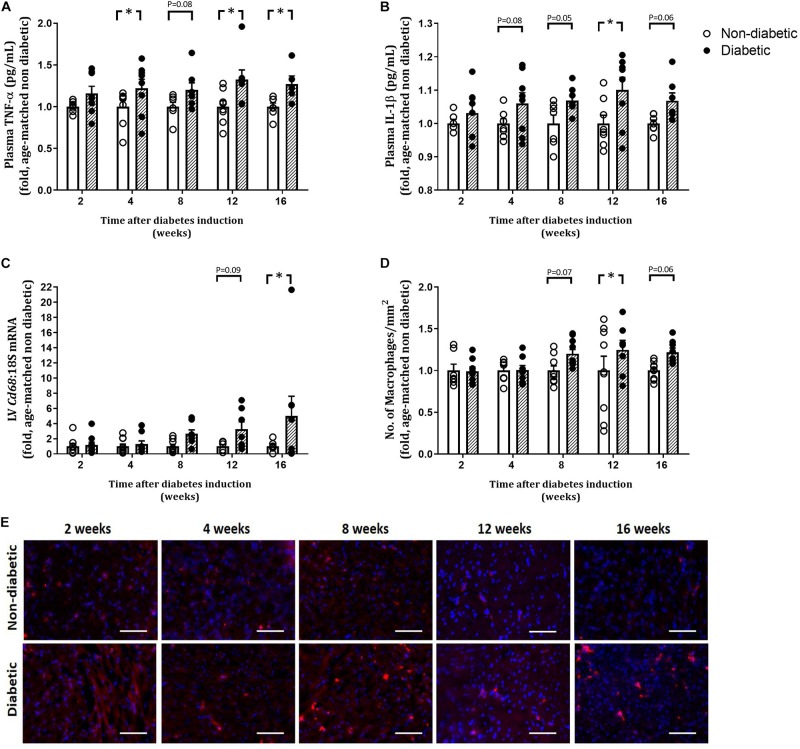
Measures of inflammation. Plasma TNFα and IL-1β were measured by ELISA **(A,B)**. LV gene expression of *Cd68* is shown **(C)**. CD68 positive cell count and representative images for CD68 macrophage stained red/pink displayed at each endpoint is shown **(D,E)**. Scale bar = 100 μm. Data are presented as mean ± SEM. *P < 0.05 vs age-matched non-diabetic mice (two-way ANOVA with Benjamini and Hochberg *post hoc* test). *n* = 5–10/group (refer to Supplementary Figure S1).

The number of total white blood cells (WBCs) per mouse was unaltered by diabetes (Table 2). The number of monocytes (as a percentage of WBC) was increased overall by diabetes (*P* = 0.004) and was increased at 4, 8, and 16 weeks of diabetes on *post hoc* analysis (*P* < 0.05 for all, Table 2). Levels of neutrophils and lymphocytes (as a percentage of WBC) were unaltered with diabetes (Table 2). Both eosinophils and basophils (as a percentage of WBC) were unaltered by diabetes overall; however, each were increased at 4 weeks of diabetes on *post hoc* analysis. Circulating plasma TNFα levels were increased overall by diabetes (*P* < 0.001) and was increased from 4 weeks of diabetes (Figure 4A). Circulating plasma IL-1β was also increased overall by diabetes (*P* < 0.001), was increased at 12 weeks of diabetes (*P* < 0.05), and tended to be increased at 4, 8, and 16 weeks of diabetes on *post hoc* analysis (Figure 4B). LV gene expression of CD68 was increased overall by diabetes (*P* = 0.003) and was increased by 16 weeks of diabetes (*P* < 0.05) (Figure 4C). This was accompanied by an overall increase in LV CD68+ stained macrophages with diabetes (*P* = 0.012), an increase at 12 weeks, and a tendency to be increased at both 8 and 16 weeks of diabetes on *post hoc* analysis (*P* = 0.07 and *P* = 0.06, respectively), as measured by immunohistochemistry (Figures 4D,E).

**TABLE 2:** Circulating markers of inflammation determined at endpoint.

**Circulating markers of inflammation**	**Non-diabetic**					**Diabetic**				
**Time after diabetes induction (weeks)**	**2**	**4**	**8**	**12**	**16**	**2**	**4**	**8**	**12**	**16**
*n*	*7*	*10*	*9*	*9*	*11*	*15*	*9*	*10*	*11*	*8*
White blood cells (WBCs) (×1000 cells/μl)	2.800.39	3.530.65	2.590.27	2.390.22	2.380.27	3.130.22	3.160.48	2.470.28	2.220.27	1.810.22
Monocytes (%WBC)	4.30.5	3.60.6	3.60.7	4.20.6	4.00.5	5.40.5	6.01.1*	5.60.7*	4.00.5	7.01.2*
Neutrophils (%WBC)	11.61.7	16.92.0	17.41.5	18.52.1	11.62.3	10.62.1	15.11.5	17.43.5	17.52.4	10.41.6
Lymphocytes (%WBC)	82.21.9	79.21.9	78.31.9	76.01.6	84.12.3	82.42.1	77.32.0	76.73.3	77.62.6	82.02.5
Eosinophils (%WBC)	1.50.6	0.20.1	0.60.5	1.10.5	0.30.1	1.30.4	1.30.5*	0.30.1	0.60.2	0.60.3
Basophils (%WBC)	0.50.2	0.040.02	0.10.1	0.20.1	0.080.04	0.30.1	0.40.1*	0.010.01	0.20.1	0.20.1

Total white blood cells (WBCs) and the number of monocytes, neutrophils, lymphocytes, eosinophils, and basophils as a percentage of total WBC were measured in whole blood at end point using a hematology analyzer. Data are presented as mean ± SEM and analyzed using a two-way ANOVA with Benjamini and Hochberg *post hoc* test. *P < 0.05 vs age-matched non-diabetic mice. Italicized values are number of animals per group.

### Progression of Cardiac Remodeling

**FIGURE 5:**
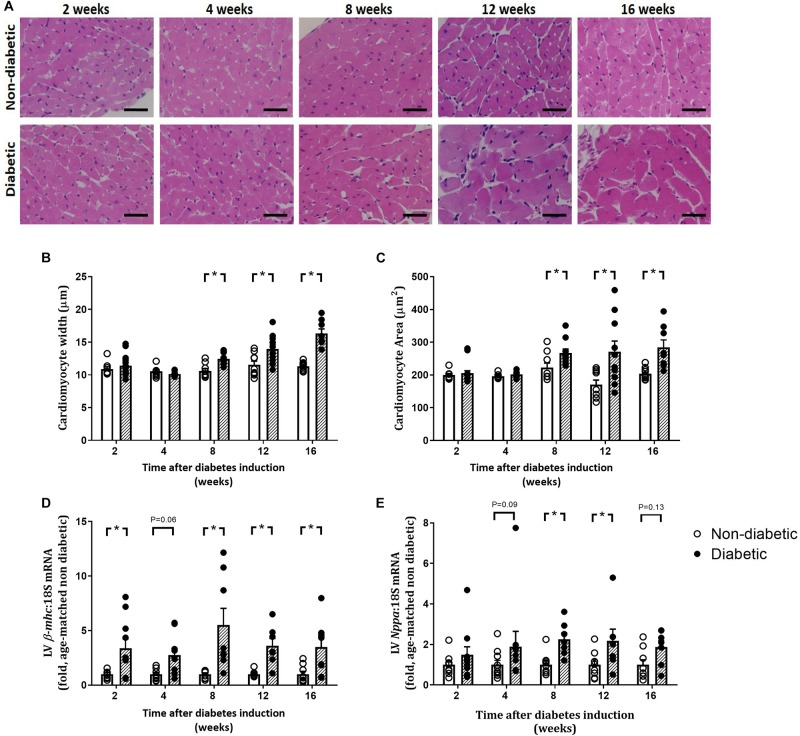
Progression of cardiomyocyte hypertrophy. Cardiomyocyte width and area, from H&E-stained LV sections **(A–C)**, gene expression of hypertrophic markers β-myosin heavy chain, β*-mhc* **(D)** and *Nppa* **(E)** are shown. Data are presented as mean ± SEM. *P < 0.05 vs age-matched non-diabetic mice (two-way ANOVA with Benjamini and Hochberg *post hoc* test). Representative H&E images **(A)** for each endpoint are shown. Scale bar = 20 μm. *n* = 7–15/group (refer to Supplementary Figure S1).

**FIGURE 6:**
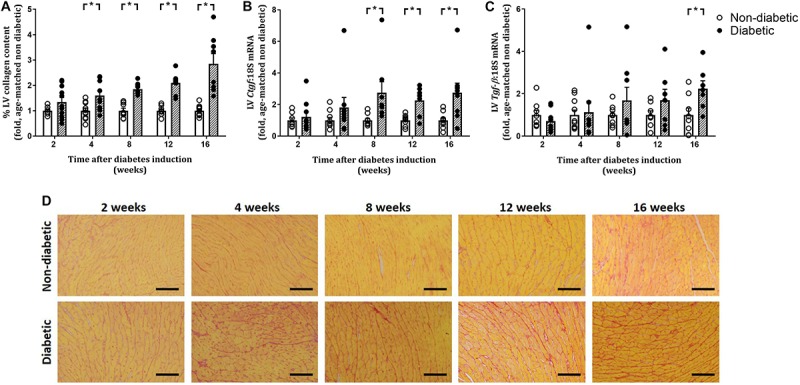
Progression of cardiac fibrosis. Percentage of picrosirius red stained collagen fibers **(A,D)** and gene expression of *Ctgf* **(B)** and *Tgf-*β **(C)** increased with the progression of diabetes. Data are presented as mean ± SEM. *P < 0.05 vs age-matched non-diabetic mice (two-way ANOVA with Benjamini and Hochberg *post hoc* test). Representative images for each endpoint are shown **(D)**. Scale bar = 40 μm. *n* = 7–11/group (refer to Supplementary Figure S1).

Left ventricular cardiomyocyte size in terms of both area and width was increased overall by diabetes (*P* < 0.001 for both) and was evident from 8 weeks of diabetes on *post hoc* analysis (*P* < 0.05) (Figures 5A–C). This was accompanied by an increase in the gene expression of hypertrophic markers β*-mhc* (overall, *P* < 0.0001 and at each time point from 2 weeks of diabetes on *post hoc* analysis, Figure 5D) and *Nppa* (overall, *P* = 0.0003 and from 8 weeks of diabetes, Figure 5E). LV fibrosis in terms of percentage of picrosirius red-stained collagen fibers was significantly increased overall with diabetes (*P* < 0.0001) and from 4 weeks of diabetes on *post hoc* analysis (*P* < 0.05, Figures 6A,D). Similarly, gene expression of the pro-fibrotic marker *Ctgf* was significantly increased overall by diabetes (*P* < 0.0001) and from 8 weeks of diabetes (*P* < 0.05) (Figure 6B), while *Tgf-*β was increased overall (*P* = 0.007) and after 16 weeks of diabetes (*P* < 0.05, Figure 6C). *Postn* tended to be increased overall by diabetes (*P* = 0.055) and on *post hoc* analysis tended to be increased at 4 weeks (*P* = 0.09) and was significantly increased by 8 weeks (*P* < 0.05) of diabetes (not shown).

### Other Markers of Diabetic Cardiomyopathy

**FIGURE 7:**
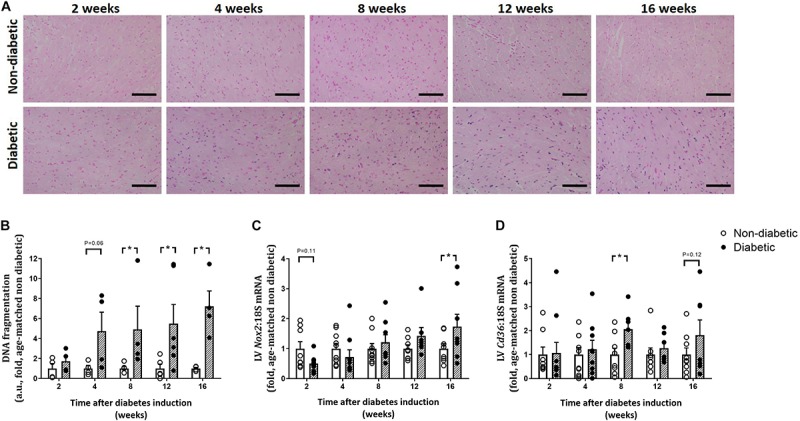
Progression of other characteristics of the diabetic heart. DNA fragmentation (cardiomyocyte apoptosis), measured by CardioTACs as the percentage of blue positive-stained apoptotic cells to non-apoptotic negatively stained red cells, was increased from 4 weeks of diabetes **(A,B)**. Representative images for each endpoint are shown **(A)**. Scale bar = 40 μm. LV gene expression of *Nox2* was increased by 16 weeks **(C)** while gene expression of the fatty acid transporter *Cd36* was increased from 8 weeks of diabetes **(D)**. Data are presented as mean ± SEM. *P < 0.05 vs age-matched non-diabetic mice (two-way ANOVA with Benjamini and Hochberg *post hoc* test). DNA fragmentation: *n* = 4–6/group; *Nox2* and *Cd36* gene expression: *n* = 7–10/group (refer to Supplementary Figure S1).

CardioTACs, a TUNEL-based assay to measure DNA fragmentation (a marker of cardiomyocyte apoptosis), was increased overall by diabetes (*P* < 0.001), tended to be increased after 4 weeks of diabetes (*P* = 0.06) and was significantly increased from 8 weeks of diabetes on *post hoc* analysis (*P* < 0.05, Figures 7A,B). LV gene expression of *Nox2* (a subunit of the ROS-generating enzyme, NADPH oxidase), was not altered by diabetes overall, however tended to be reduced early at 2 weeks of diabetes (*P* = 0.11), and was significantly increased by 16 weeks of diabetes (*P* < 0.05) (Figure 7C). Gene expression of the fatty acid transporter *Cd36* in the LV was significantly increased overall by diabetes (*P* = 0.034) and at 8 weeks of diabetes (Figure 7D).

### Progression of LV O-GlcNAcylation

**FIGURE 8:**
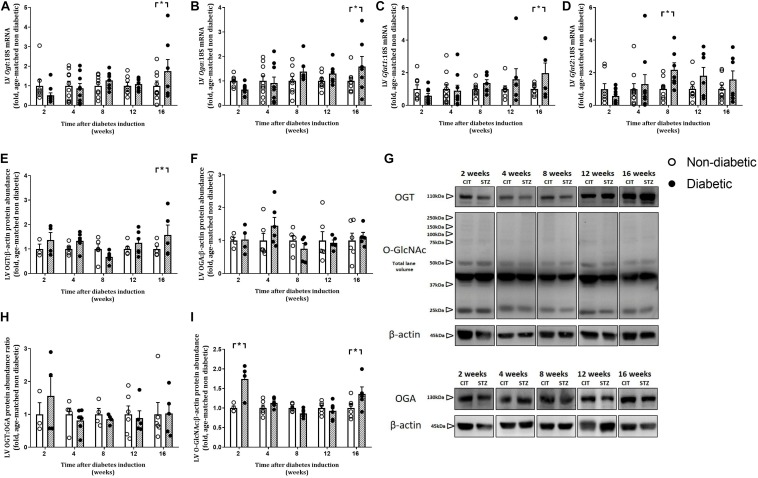
Progressive changes in LV O-GlcNAcylation and its regulating enzymes. Gene expression of O-GlcNAc transferase (*Ogt*: adds O-GlcNAc) **(A)** and O-GlcNAcase (*Oga*: removes O-GlcNAc) **(B)** and the rate limiting enzymes of the HBP (*Gfat1* and *Gfat2*, involved in O-GlcNAcylation) **(C,D)** involved in O-GlcNAcylation were increased with diabetes. LV protein abundance of OGT **(E,G)**, OGA **(F,G)**, OGT:OGA protein abundance ratio **(H)**, and total LV O-GlcNAcylated proteins **(I,G)** were measured in the heart at endpoint (using the CTD110.6 antibody for O-GlcNAc), and normalized to β-actin. Note the multiple bands in the O-GlcNAc blot including multiple faint high molecular weight bands. Data are presented as mean ± SEM. *P < 0.05 vs age-matched non-diabetic mice (two-way ANOVA with Benjamini and Hochberg *post hoc* test). O-GlcNAc/OGT western blots: *n* = 3–6/group; gene expression: *n* = 7–10/group (refer to Supplementary Figure S1). For all raw uncut western blots refer to Supplementary Presentation.

Left ventricular gene expression of the two regulatory enzymes involved in O-GlcNAcylation, *Ogt* and *Oga*, were significantly increased only at 16 weeks of diabetes (*P* < 0.05 for both, Figures 8A,B). LV gene expression of the *Gfat1* isoform of the rate-limiting enzyme of the HBP was increased only at 16 weeks of diabetes (Figure 8C). LV gene expression of the other isoform, *Gfat2*, was increased by diabetes overall (*P* = 0.041) and was increased at 8 weeks of diabetes (Figure 8D). Protein abundance of OGT tended to be increased overall by diabetes (*P* = 0.072) and was also increased at 16 weeks of diabetes on *post hoc* analysis (*P* < 0.05, Figures 8E,G). Protein abundance of OGA and the OGT:OGA ratio was not altered by diabetes or at any age (Figures 8F,H,G). Total LV O-GlcNAcylated protein level, analyzed by total lane volume, was increased overall by diabetes (*P* = 0.003) and on *post hoc* analysis was increased early at 2 weeks of diabetes and then again at 16 weeks of diabetes (Figures 8I,G).

## Discussion

This study has described the progression of diabetic cardiomyopathy, the multifactorial nature of its development, and revealed new insights into the relative timing of these factors. We confirm that hyperglycemia in this mouse model of T1D, as one of the earliest changes, may be the central driver in the development of diabetic cardiomyopathy. The decline in LV diastolic function as diabetes progressed was accompanied by systemic inflammation and an increase in the LV macrophage content. Increases in the extent of each of cardiomyocyte hypertrophy, apoptosis, interstitial fibrosis, inflammation, and oxidative stress all increased in the myocardium as diabetes progressed, likely as a consequence of cardiac macrophage infiltration. In addition, increases in expression and activity of components of the HBP, an alternate fate of glucose metabolism, was also observed with diabetes progression.

### LV Diastolic and Systolic Dysfunction Worsens With Diabetes Progression

The development of systolic dysfunction and heart failure in diabetic cardiomyopathy has been shown to occur after the initial manifestation of LV diastolic dysfunction, indicative of impaired cardiac relaxation (Huynh et al., 2014). The E/A ratio is typically used as a marker of LV diastolic function and is reduced in the setting of diabetes mainly due to altered cardiac remodeling. Since the first study to characterize diabetic cardiomyopathy in diabetic patients in 1972, most human and animal studies have demonstrated that this relationship is primarily associated with the onset of diastolic dysfunction (Rubler et al., 1972; Wold et al., 2005). Previous studies in our laboratory in this same mouse model of T1D demonstrated diastolic dysfunction, in terms of reduced E/A and increased peak A wave velocity, deceleration time, and IVRT, is evident at 8 weeks of diabetes; however, now we reveal that some of these key markers of diastolic function are impaired earlier than we had previously reported (Huynh et al., 2010; De Blasio et al., 2015; Prakoso et al., 2017). We can now confirm that a reduction in LV diastolic function, as measured by Doppler echocardiography, is evident from 4 weeks of diabetes induction, as highlighted by increased peak A-wave velocity and a reduced E/A ratio (which then progressively worsen with diabetes). This is accompanied by progressive increases in deceleration time and IVRT, and a reduction in FS, with disease progression from 8 weeks of diabetes onward. This reduction in diastolic and systolic function from 4 weeks after diabetes induction may be due to altered cardiac contraction and intracellular calcium handling. This has been demonstrated to be impaired in isolated ventricular myocytes of mice after a short-term STZ-induced diabetes (2 weeks) (Norby et al., 2004; Ceylan-Isik et al., 2006a). Interestingly, this can be ameliorated by cardiac overexpression of IGF-1 (Norby et al., 2004). In a study of Zucker rats with long-term diabetes, there is evidence of cardiac structural and functional changes at 16 weeks of age with a progressive decline in end diastolic volume, end diastolic pressure, and end systolic volume from 16 to 36 weeks of age (Baynes and Murray, 2009). In humans, cardiac structure and function were measured in adults with T1D 4 years after an initial echocardiogram. The study showed that one in five had a normal first cardiac echocardiogram, however, had developed an abnormal second echocardiogram with progressive changes such as a reduced peak septal mitral annular systolic velocity (S′), increased A wave velocity, increased deceleration time, and a reduced e’ and E/e’ ratio (Wai et al., 2014). In patients with T2D, age is the strongest predictor of declining cardiac function with the risk of an abnormal echocardiogram increasing by 9% for each year over 50 years of age (Srivastava et al., 2008). The potential mechanisms leading to this progressive decline in diastolic function remain to be determined.

### LV Remodeling Worsens With Diabetes Progression

Impaired cardiac function in terms of LV diastolic dysfunction is associated with interstitial fibrosis and pathological cardiomyocyte hypertrophy in animal models of diabetes (Huynh et al., 2014; Marwick et al., 2018), a pathological adaption to diabetes that is linked to increased expression of pro-fibrotic *Tgf-*β (Mizushige et al., 2000). In the present study, the reduction in diastolic function may be partially attributable to the increase in cardiac collagen deposition observed in diabetic mice, resulting in increased LV wall stiffness. We report that LV collagen deposition was increased by 4 weeks of diabetes, and progressively worsens up to 16 weeks of diabetes (as measured by histology). This is accompanied by early and sustained increases in gene expression of pro-fibrotic markers *Tgf-*β, *Ctgf*, and *Postn* from 8 weeks of diabetes.

Pathological LV hypertrophy is often evident in patients with diabetes and is a clinical marker for heart failure (Bernardo et al., 2010; Huynh et al., 2014). Animal models of heart failure show that pathological signaling pathways are upregulated and are also associated with a decline in viable myocytes (Bernardo et al., 2010). In this study, we show that changes in myocyte size are evident by 8 weeks of diabetes in this model, in terms of cardiomyocyte width and area, coinciding with an increase in gene expression of the pro-hypertrophic marker *Nppa*. Surprisingly, this was preceded by an early increase in gene expression of the pro-hypertrophic marker β*-mhc*, from 2 weeks of diabetes. Additionally, cardiomyocyte apoptosis, measured by an increase in DNA fragmentation, accompanied this cardiac remodeling in terms of fibrosis and hypertrophy from 4 weeks of diabetes. This supports many previous studies that have shown that cardiomyocyte apoptosis is correlated with hyperglycemia and is associated with cardiac remodeling seen in diabetic cardiomyopathy (Fiordaliso et al., 2000; Frustaci et al., 2000; Cai et al., 2002; Ritchie et al., 2012; Huynh et al., 2013).

### LV Oxidative and ER Stress Increases With Diabetes Progression

For many years, oxidative stress, the result of an imbalance of ROS production and antioxidant status, has been implicated in the development and progression of diabetic cardiomyopathy (Wold et al., 2005; Huynh et al., 2013; Prakoso et al., 2017). The increase in ROS generation observed in the diabetic heart results in cardiac damage, remodeling, and altered function (Ritchie, 2009). Previously in this model of T1D, we have shown that the expression of LV NADPH oxidase subunits p47^phox^ and Nox2, along with increases in superoxide levels (measured by lucigenin chemiluminescence), gene expression of *Nox4* and protein abundance of PKCβ2, were significantly upregulated after 12–14 weeks of diabetes (Huynh et al., 2013; Prakoso et al., 2017). Our results here support these previous findings in terms of gene expression of *Nox2*, which was increased after 16 weeks of diabetes. A previous study has shown that cardiac oxidative stress was increased earlier at 4 weeks after STZ-induced diabetes induction, measured as a reduction in the GSH-to-GSSG ratio and an increase in AGE formation (Ceylan-Isik et al., 2006b).

### LV Macrophage Infiltration and Inflammation Increase With Diabetes Progression

Diabetes is regarded as a pro-inflammatory disease state that is associated with elevated levels of inflammatory mediators which can lead to a range of chronic pathologies including cardiomyopathy. Elevated levels of cytokines are present in the heart of animal models of diabetes and serum of diabetic patients (Azar et al., 2000; Torre-Amione, 2005; Tschope et al., 2005; Ares-Carrasco et al., 2009). We show that plasma levels of TNFα and IL-1β are increased early, and persist from 4 weeks of diabetes. This is accompanied by increases in LV gene expression of *Tnf*α, concomitantly with the macrophage marker *Cd68* and increased macrophage content in the diabetic heart from 8 to 12 weeks of diabetes. The increase in these cytokines with diabetes supports previous observations of increases in TNFα, IL-1β, and TGFβ, and concomitant increases in fibrosis and diastolic dysfunction, in mice with diabetes (Westermann et al., 2006; Ares-Carrasco et al., 2009).

### Impact of Diabetes Progression on LV HBP/O-GlcNAcylation

The adverse outcomes evident in the diabetic heart have been linked to a sustained (and likely maladaptive) increase in protein O-GlcNAc levels (Wright et al., 2017). We have shown that the HBP is upregulated with diabetes. We show that gene expression of both isoforms of *Gfat*, the rate-limiting enzyme of the HBP, is increased from around 8 weeks of diabetes. This is accompanied by an increase in the LV gene expression of *Oga* and *Ogt*, and LV protein abundance of OGT at 16 weeks of diabetes, while OGA protein abundance was unaltered. Interestingly, the impact of T1D on LV O-GlcNAcylation appeared to be biphasic, with an early increase evident at 2 weeks (shortly after the initiation of hyperglycemia) and then a second increase evident later, at 16 weeks of diabetes (concomitant with maximal cardiomyopathy phenotype in the diabetic mouse heart). We thus speculate that the early increase in O-GlcNAcylation and may be a compensatory mechanism to counteract the increase in circulating glucose concentrations due to the induction of diabetes, while the later increase may be indicative that the system has been overwhelmed and becomes detrimental. Many proteins have been shown to be O-GlcNAcylated in the setting of diabetes, altering their function and contributing to the resultant detrimental outcomes of the disease (Marsh et al., 2014). The relationship between cardiac O-GlcNAc levels and development of cardiomyopathy in terms of hypertrophy and fibrosis is currently unclear. Previous studies have suggested that sustained O-GlcNAcylation in the heart may be pro-hypertrophic and pro-fibrotic since cardiomyocyte-specific knockout of OGT in mice results in increases in cardiomyocyte size and fibrosis (Watson et al., 2010, 2014). However, others have suggested that increased protein O-GlcNAcylation (subsequent to upregulation of the HBP) may conversely blunt hypertrophic signaling in diabetic cardiomyocytes within 24 h of treatment with either Angiotensin II or phenylephrine; inhibition of the HBP in this setting partially restored hypertrophic signaling responses (Marsh et al., 2011). Here we show that cardiomyocyte size increases from 8 weeks of diabetes and coincides with the increase in GFAT expression but not protein levels of O-GlcNAcylation, which increase later after the induction of diabetes.

### Limitations and Considerations

We utilized the STZ-induced mouse model of T1D to assess the progression of diabetic cardiomyopathy. The scope of this study was to determine this in male mice which is a clear limitation. We have recently shown that diastolic dysfunction is more profound in STZ-induced diabetic female mice despite less pronounced hyperglycemia (Chandramouli et al., 2018). Therefore, determination of the progression of markers of cardiomyopathy should be extended to females in future studies given that cardiovascular risk is greater in women (Marwick et al., 2018). This may reveal that females exhibit even earlier alterations in markers of diabetes-induced cardiomyopathy as measured in this study. Here we have determined some of the major contributors to the development of cardiomyopathy in the setting of diabetes; however, there are other major contributing pathways (e.g., mitochondrial function, calcium handling) that remain to be determined.

This is the first study to our knowledge to describe the time-dependent progression of markers contributing to the development of diabetic cardiomyopathy in a mouse model of T1D. We can confirm that multiple pathways lead and contribute to diabetes progression at varying time points after the initial insult. Time points of therapeutic intervention for future studies in animal models of type 1 diabetes should select the timing of their end points judiciously, depending on whether their goal is to prevent or reverse the cardiac complications of the disease.

## Data Availability Statement

The datasets generated for this study are available on request to the corresponding author.

## Ethics Statement

All animal research was conducted in accordance with the “National Health and Medical Research Council of Australia” guidelines, and was approved by the Alfred Research Alliance (ARA) Animal Ethics committee (AEC Ethics approval#: E/1535/2015/B).

## Author Contributions

MJD and RR contributed to study conception and design. MJD, NH, MD, JW, AW, LD, DD, DP, and HK performed the experiments. MJD, NH, LD, HK, DD, and RR analyzed the data. MJD, JC, and RR interpreted the results. MJD prepared figures and drafted the manuscript. All authors edited and revised the manuscript.

## Conflict of Interest

The authors declare that the research was conducted in the absence of any commercial or financial relationships that could be construed as a potential conflict of interest.

## Footnotes

1 ELISAkit.com

## Supplementary Material

FIGURE S1 Flow diagram of animal use and analysis based on the CONsolidated Standards of Animal Experiment ReporTing (CONSAERT) template.

FIGURE S2 Organ weights relative to body weight. Data are presented as mean ± SEM. **P* < 0.05 vs age-matched non-diabetic mice (two-way ANOVA with Benjamini and Hochberg *post hoc* test). *n* = 8–16/group (refer to Supplementary Figure S1).

FIGURE S3 Echocardiography data relative to normalized age-matched non-diabetic controls for each time point. Data are presented as mean ± SEM. **P* < 0.05 vs age-matched non-diabetic mice (two-way ANOVA with Benjamini and Hochberg *post hoc* test). *n* = 7–11/group (refer to Supplementary Figure S1).

PRESENTATION All raw uncut western blots for Figure 8 are presented.
