# Supplementary figures and images for: Defining the Progression of Diabetic Cardiomyopathy in a Mouse Model of Type 1 Diabetes
Source: Front Physiol. 2020 Feb 20;11:124. doi: 10.3389/fphys.2020.00124 (PMC7045054; doi:10.3389/fphys.2020.00124)

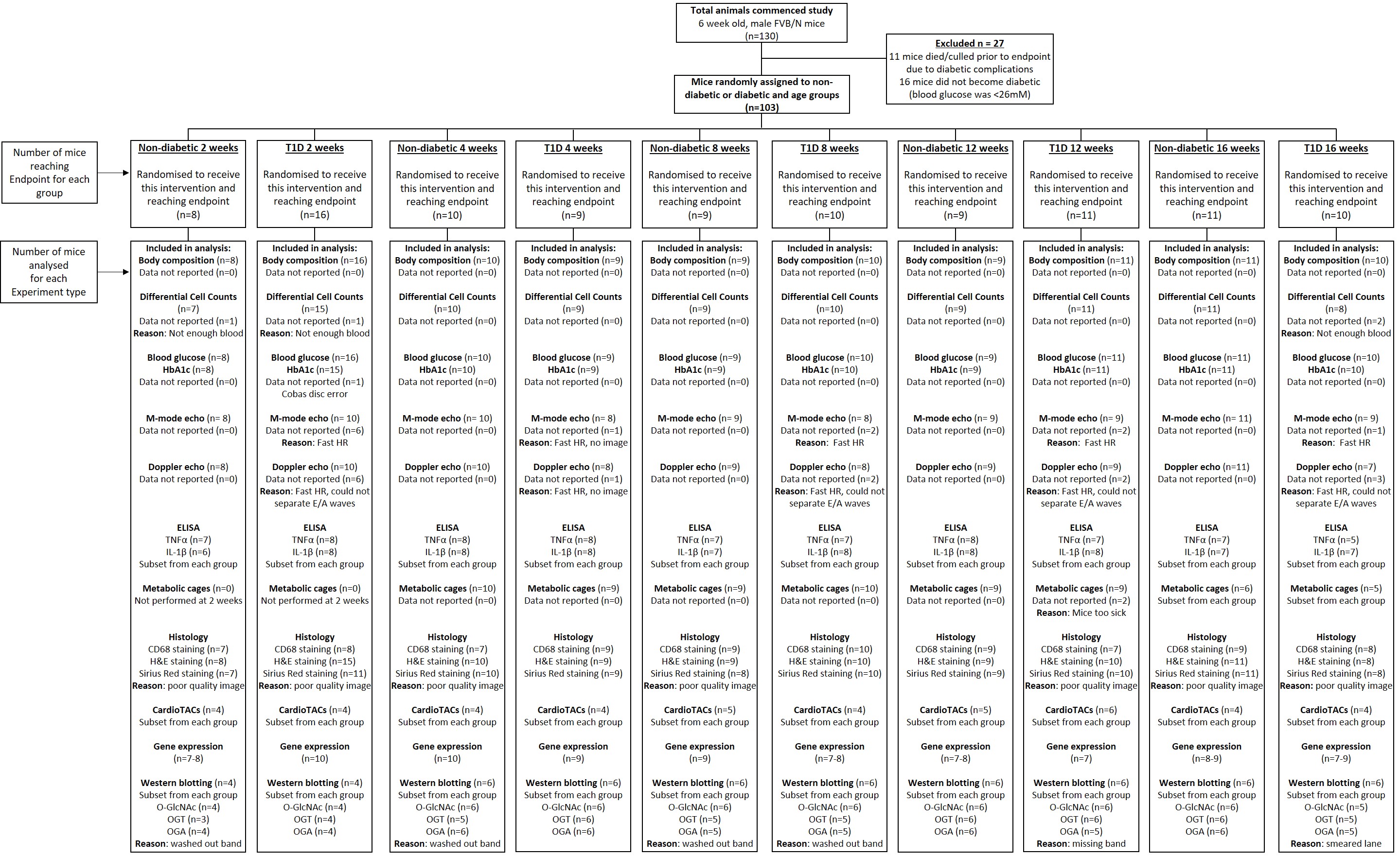

Supplement: FIGURE S1 — Flow diagram of animal use and analysis based on the CONsolidated Standards of Animal Experiment ReporTing (CONSAERT) template. [file Image_1.JPEG]

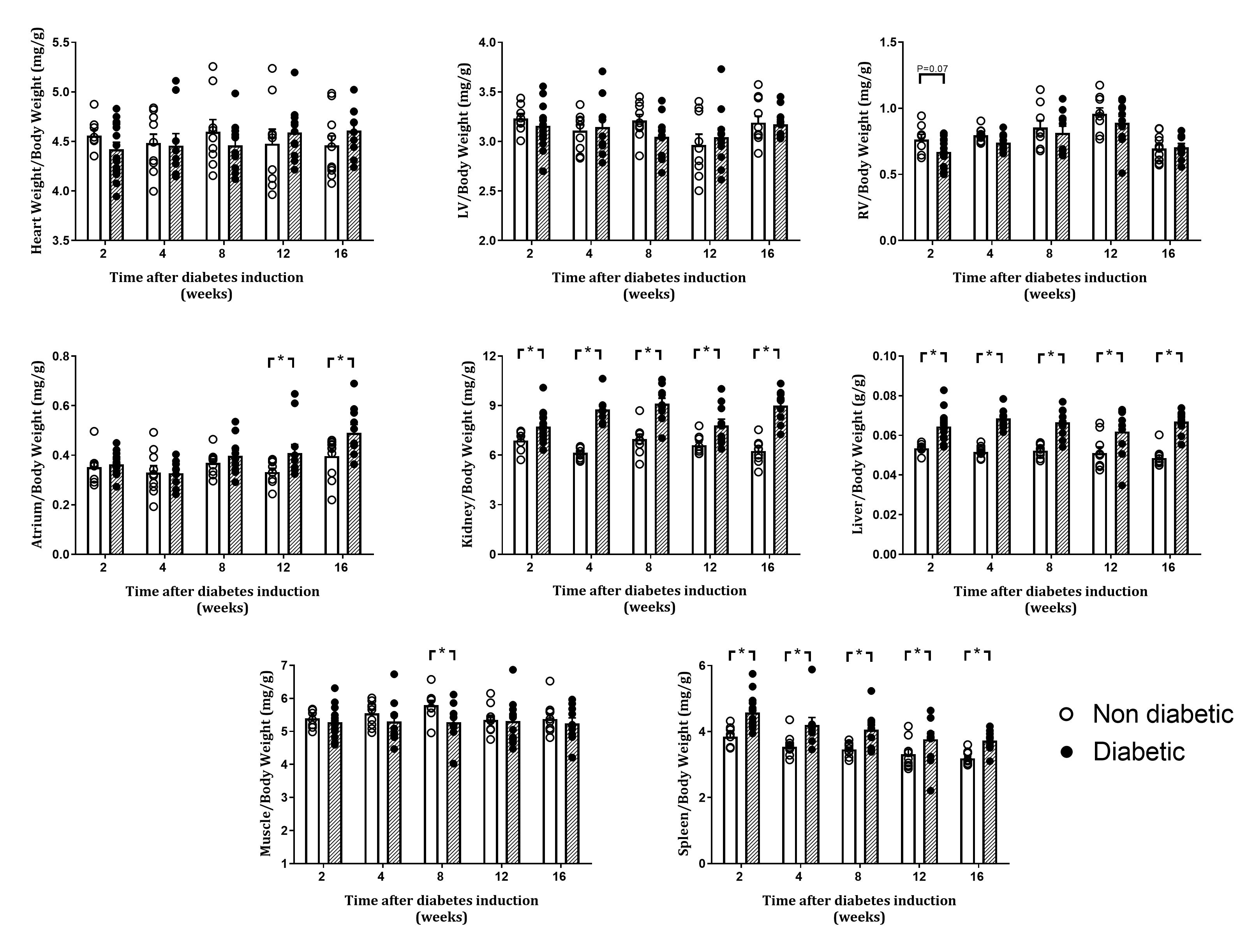

Supplement: FIGURE S2 — Organ weights relative to body weight. Data are presented as mean ± SEM. *P < 0.05 vs age-matched non-diabetic mice (two-way ANOVA with Benjamini and Hochberg post hoc test). n = 8–16/group (refer to Supplementary Figure S1). [file Image_2.JPEG]

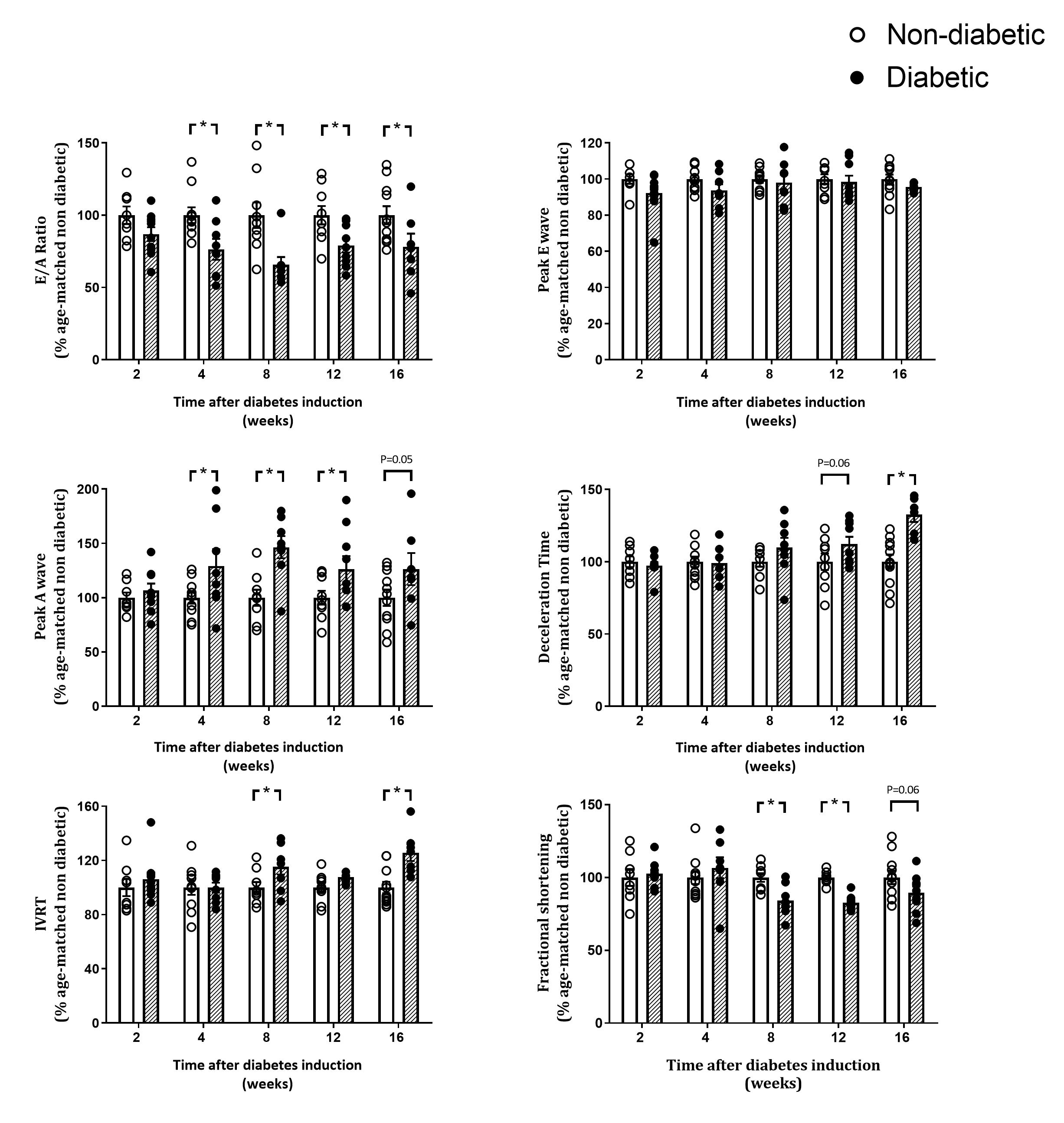

Supplement: FIGURE S3 — Echocardiography data relative to normalized age-matched non-diabetic controls for each time point. Data are presented as mean ± SEM. *P < 0.05 vs age-matched non-diabetic mice (two-way ANOVA with Benjamini and Hochberg post hoc test). n = 7–11/group (refer to Supplementary Figure S1). [file Image_3.JPEG]
