# Supplementary material for: Defining the Progression of Diabetic Cardiomyopathy in a Mouse Model of Type 1 Diabetes
Source: Front Physiol. 2020 Feb 20;11:124. doi: 10.3389/fphys.2020.00124 (PMC7045054; doi:10.3389/fphys.2020.00124)
Supplement: PRESENTATION — All raw uncut western blots for Figure 8 are presented. [file Presentation_1.PPTX]

## Slide 1
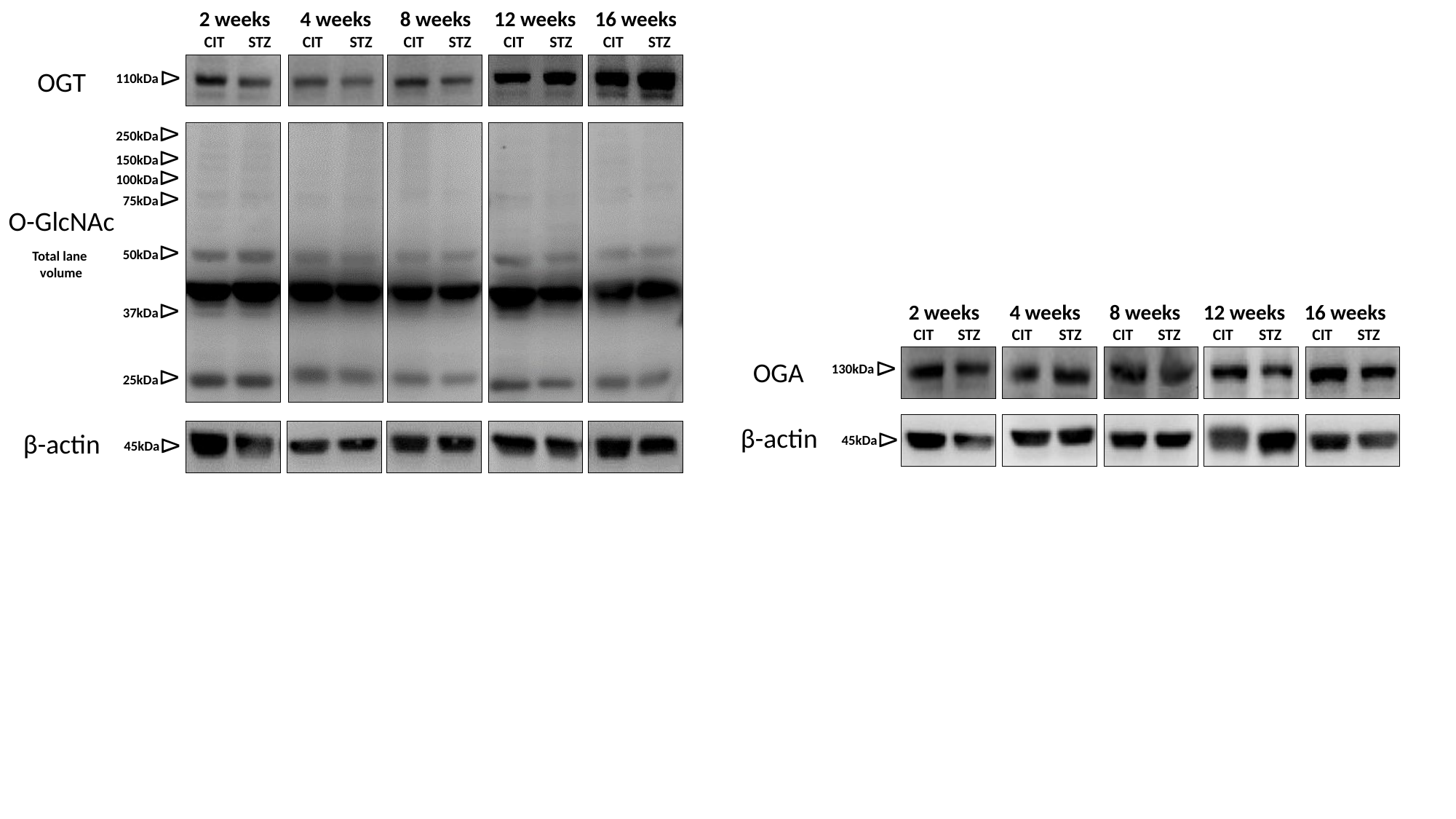

2 weeks
4 weeks
8 weeks
12 weeks
16 weeks
CIT
STZ
CIT
STZ
CIT
STZ
CIT
STZ
CIT
STZ
∆
OGT
110kDa
O-GlcNAc
Total lane
volume
β-actin
∆
45kDa
∆
250kDa
∆
150kDa
∆
100kDa
∆
75kDa
∆
50kDa
∆
37kDa
∆
25kDa
2 weeks
4 weeks
8 weeks
12 weeks
16 weeks
CIT
STZ
CIT
STZ
CIT
STZ
CIT
STZ
CIT
STZ
∆
OGA
130kDa
β-actin
∆
45kDa

## Slide 2
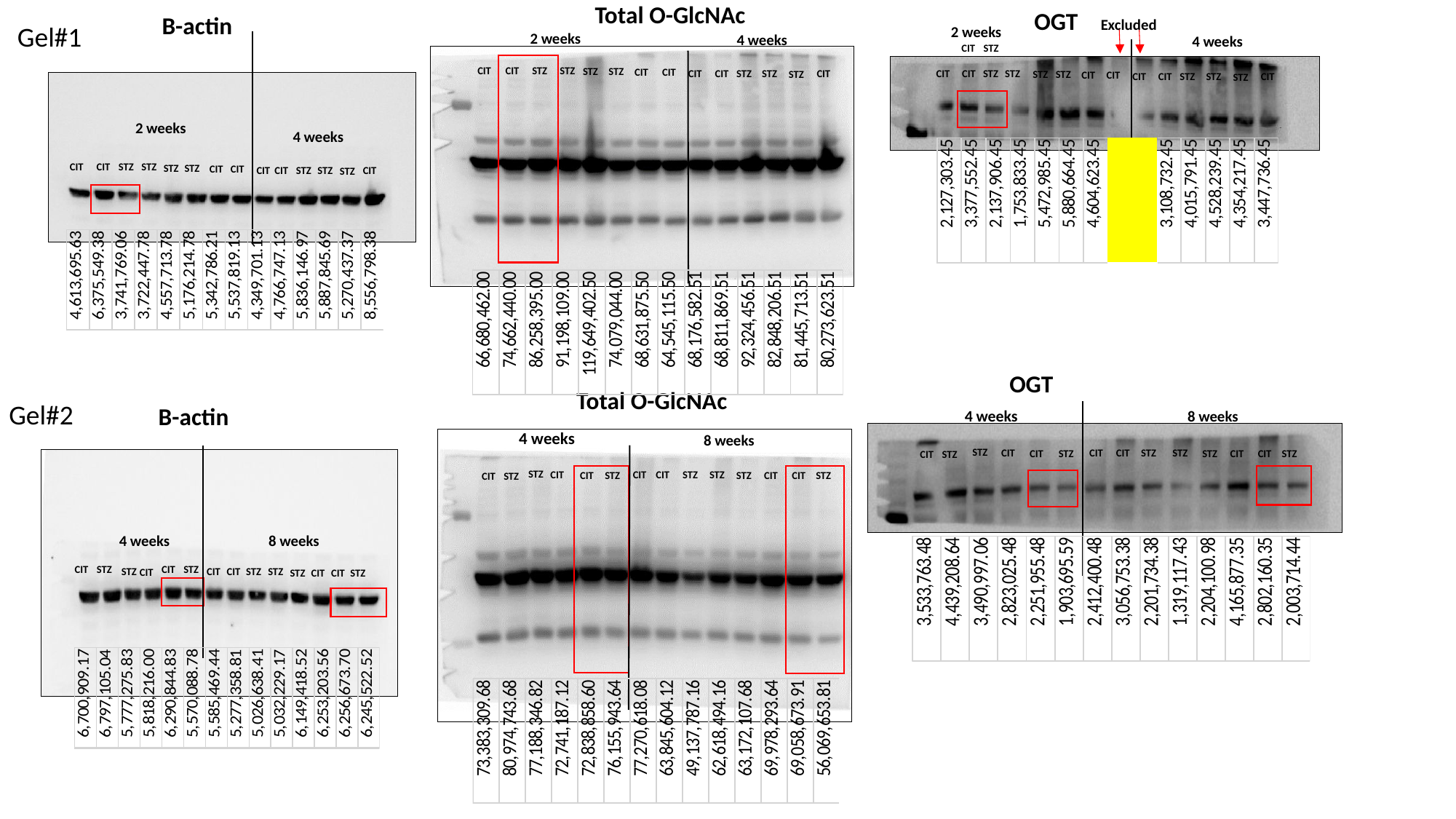

Total O-GlcNAc
OGT
B-actin
Excluded
Gel#1
2 weeks
2 weeks
4 weeks
4 weeks
CIT
STZ
STZ
CIT
STZ
CIT
STZ
STZ
CIT
CIT
STZ
CIT
STZ
CIT
STZ
STZ
CIT
CIT
CIT
STZ
STZ
STZ
CIT
CIT
STZ
STZ
CIT
CIT
CIT
STZ
2 weeks
4 weeks
STZ
CIT
STZ
CIT
STZ
STZ
CIT
CIT
STZ
STZ
CIT
CIT
CIT
STZ
OGT
Total O-GlcNAc
Gel#2
B-actin
4 weeks
8 weeks
4 weeks
8 weeks
STZ
STZ
CIT
STZ
CIT
CIT
CIT
STZ
STZ
CIT
STZ
CIT
CIT
STZ
STZ
STZ
CIT
STZ
CIT
CIT
CIT
STZ
STZ
CIT
STZ
CIT
CIT
STZ
4 weeks
8 weeks
CIT
STZ
CIT
STZ
STZ
STZ
CIT
STZ
CIT
CIT
STZ
CIT
STZ
CIT

## Slide 3
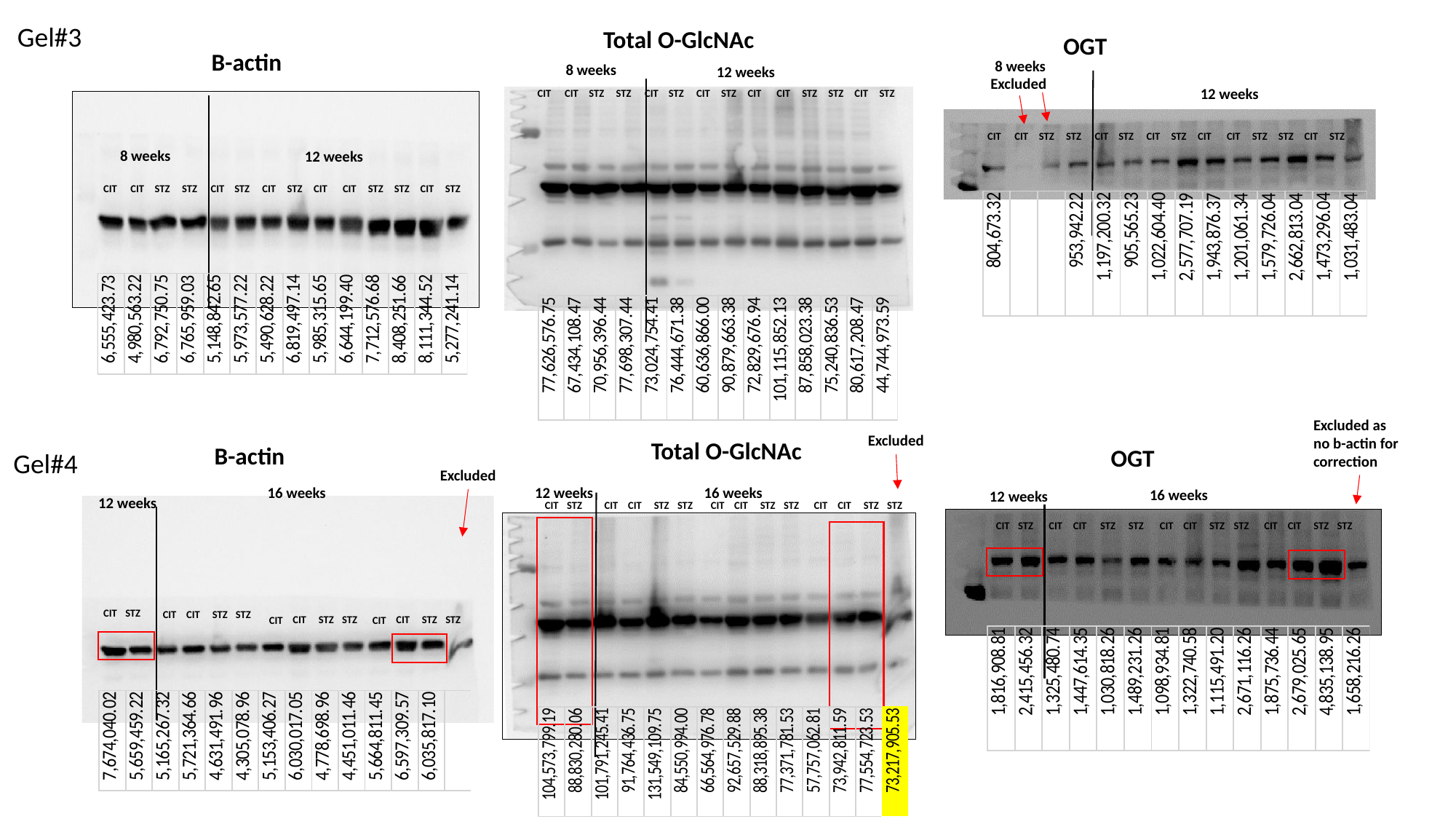

Gel#3
Total O-GlcNAc
OGT
B-actin
8 weeks
8 weeks
12 weeks
Excluded
12 weeks
CIT
CIT
STZ
STZ
CIT
STZ
CIT
STZ
CIT
CIT
STZ
STZ
CIT
STZ
CIT
CIT
STZ
STZ
CIT
STZ
CIT
STZ
CIT
CIT
STZ
STZ
CIT
STZ
8 weeks
12 weeks
CIT
CIT
STZ
STZ
CIT
STZ
CIT
STZ
CIT
CIT
STZ
STZ
CIT
STZ
Excluded as
no b-actin for correction
Excluded
Total O-GlcNAc
B-actin
OGT
Gel#4
Excluded
16 weeks
12 weeks
16 weeks
16 weeks
12 weeks
12 weeks
CIT
STZ
CIT
CIT
STZ
STZ
CIT
CIT
STZ
STZ
CIT
CIT
STZ
STZ
CIT
STZ
CIT
CIT
STZ
STZ
CIT
CIT
STZ
STZ
CIT
CIT
STZ
STZ
CIT
STZ
CIT
STZ
STZ
CIT
CIT
STZ
STZ
CIT
STZ
STZ
CIT
CIT

## Slide 4
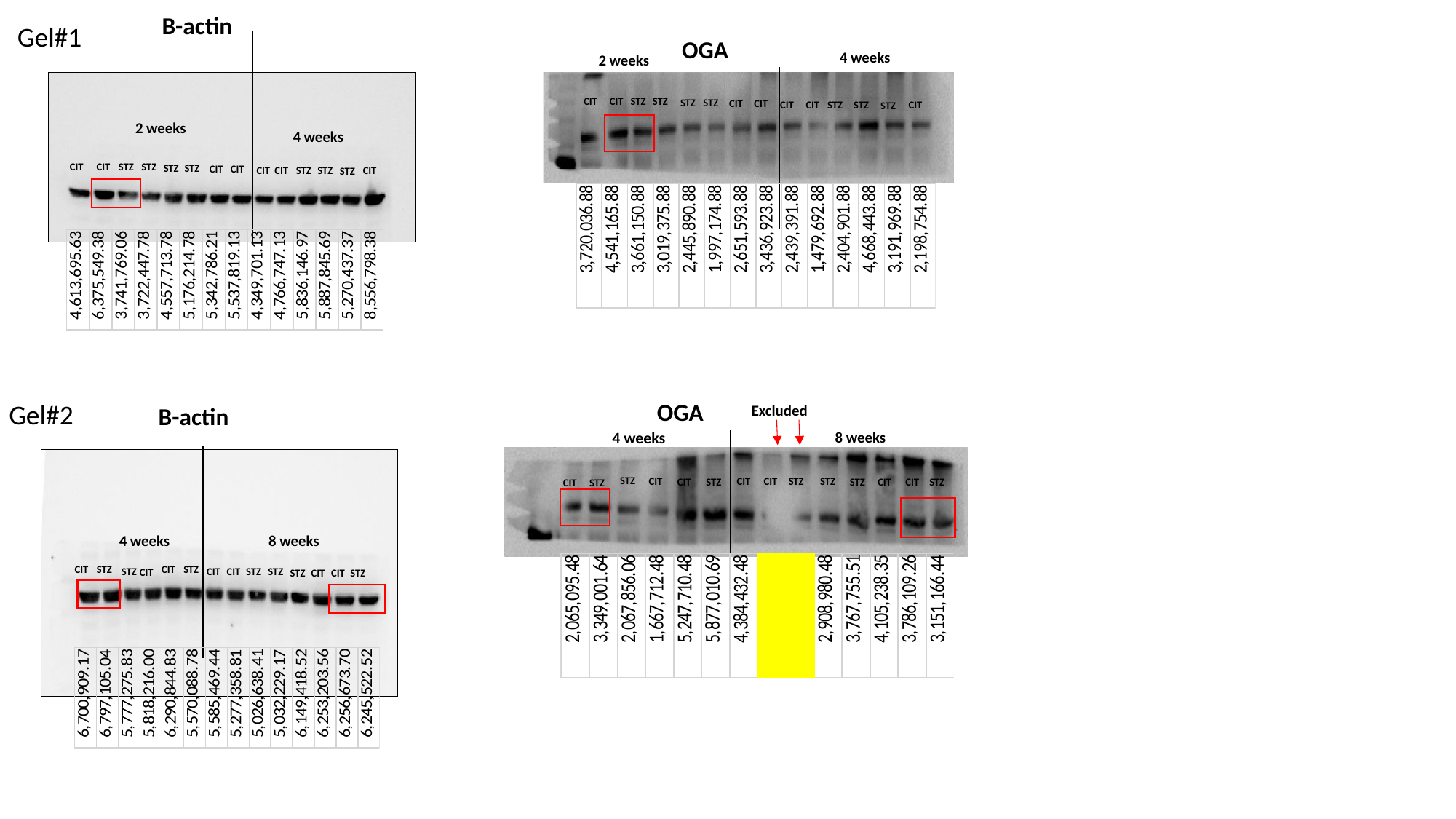

B-actin
Gel#1
OGA
4 weeks
2 weeks
STZ
CIT
STZ
CIT
STZ
STZ
CIT
CIT
STZ
STZ
CIT
CIT
CIT
STZ
2 weeks
4 weeks
STZ
CIT
STZ
CIT
STZ
STZ
CIT
CIT
STZ
STZ
CIT
CIT
CIT
STZ
OGA
Gel#2
Excluded
B-actin
4 weeks
8 weeks
STZ
STZ
CIT
STZ
CIT
CIT
CIT
STZ
STZ
CIT
STZ
CIT
CIT
STZ
4 weeks
8 weeks
CIT
STZ
CIT
STZ
STZ
STZ
CIT
STZ
CIT
CIT
STZ
CIT
STZ
CIT

## Slide 5
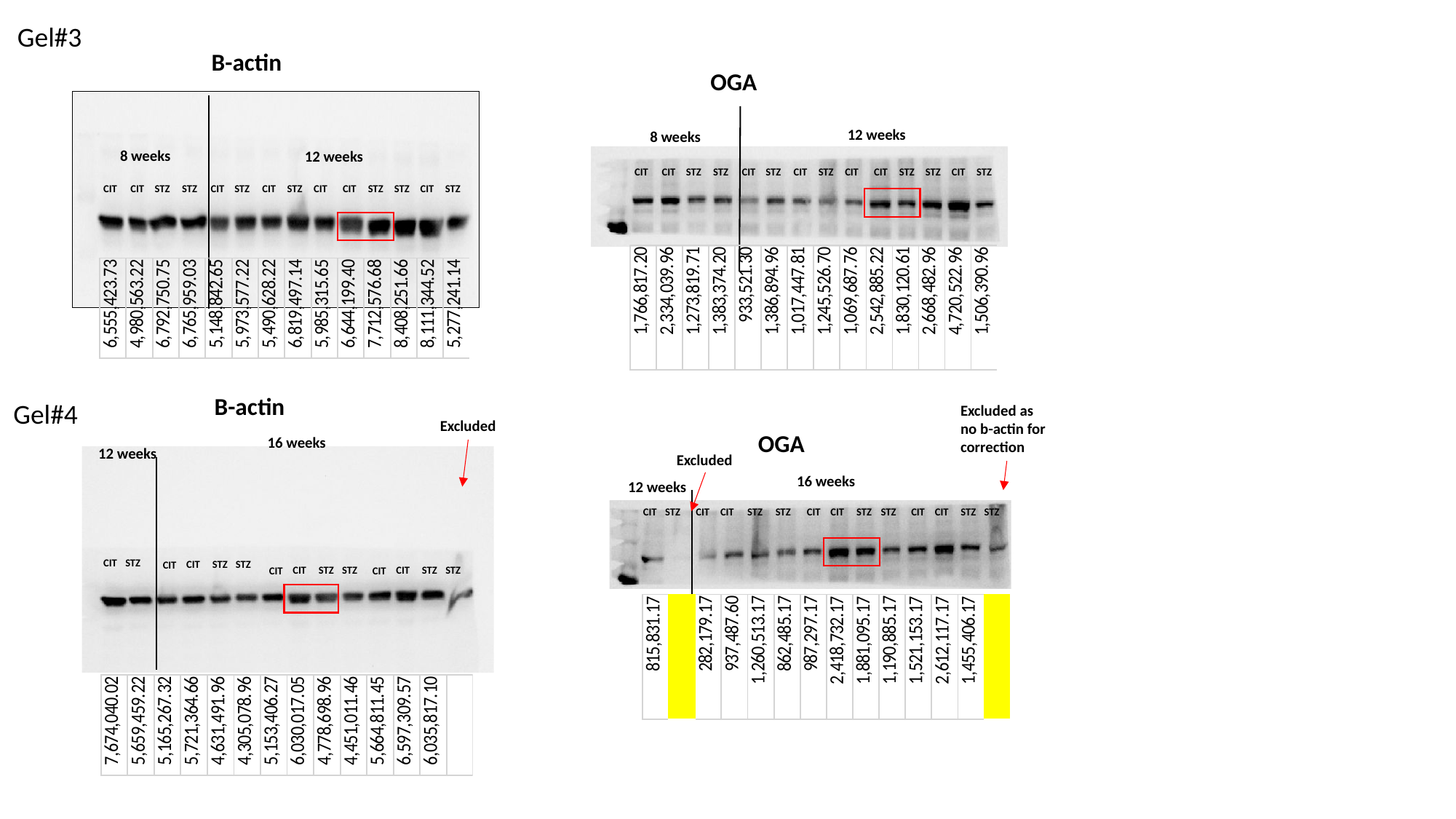

Gel#3
B-actin
OGA
12 weeks
8 weeks
8 weeks
12 weeks
CIT
CIT
STZ
STZ
CIT
STZ
CIT
STZ
CIT
CIT
STZ
STZ
CIT
STZ
CIT
CIT
STZ
STZ
CIT
STZ
CIT
STZ
CIT
CIT
STZ
STZ
CIT
STZ
B-actin
Gel#4
Excluded as
no b-actin for correction
Excluded
OGA
16 weeks
12 weeks
Excluded
16 weeks
12 weeks
CIT
STZ
CIT
CIT
STZ
STZ
CIT
CIT
STZ
STZ
CIT
CIT
STZ
STZ
CIT
STZ
CIT
STZ
STZ
CIT
CIT
STZ
STZ
CIT
STZ
STZ
CIT
CIT
